# Spatiotemporal Access Model Based on Reputation for the Sensing Layer of the IoT

**DOI:** 10.1155/2014/671038

**Published:** 2014-08-06

**Authors:** Yunchuan Guo, Lihua Yin, Chao Li, Junyan Qian

**Affiliations:** ^1^Institute of Information Engineering, Chinese Academy of Sciences, Beijing 100093, China; ^2^Beijing Key Laboratory of IOT Information Security, Beijing 100093, China; ^3^Guangxi Key Laboratory of Trusted Software, Guilin University of Electronic Technology, Guilin 541004, China

## Abstract

Access control is a key technology in providing security in the Internet of Things (IoT). The mainstream security approach proposed for the sensing layer of the IoT concentrates only on authentication while ignoring the more general models. Unreliable communications and resource constraints make the traditional access control techniques barely meet the requirements of the sensing layer of the IoT. In this paper, we propose a model that combines space and time with reputation to control access to the information within the sensing layer of the IoT. This model is called spatiotemporal access control based on reputation (STRAC). STRAC uses a lattice-based approach to decrease the size of policy bases. To solve the problem caused by unreliable communications, we propose both nondeterministic authorizations and stochastic authorizations. To more precisely manage the reputation of nodes, we propose two new mechanisms to update the reputation of nodes. These new approaches are the authority-based update mechanism (AUM) and the election-based update mechanism (EUM). We show how the model checker UPPAAL can be used to analyze the spatiotemporal access control model of an application. Finally, we also implement a prototype system to demonstrate the efficiency of our model.

## 1. Introduction

As a dynamic global ubiquitous network, the Internet of Things (IoT) links physical and virtual objects by integrating sensors, smart terminals, and global positioning systems (GPSs). Authoritative institutes predicate that the IoT will create hundreds of billions of dollars in savings and productivity gains for businesses, governments, and households: Cisco believes that the IoT will create a US$14.4 trillion business opportunity in 2020 (http://www.eetimes.com/document.asp?doc_id=1263115) and Groupe Speciale Mobile Association (GSMA) predicts that, in 2020, the connected life (one part of the IoT) will bring a US$4.5-trillion global impact on people and businesses (http://www.gsma.com/newsroom/gsma-announces-the-business-impact-of-connected-devices-could-be-worth-us4-5-trillion-in-2020/).

Along with the increasingly rapid development of the IoT, security issues have also become increasingly serious, especially when industrial controllers are either directly or indirectly connected to IoT. A typical example of this type of security compromise is the worm Stuxnet. Known as the first cyber-warfare weapon, Stuxnet was used to attack the Natanz uranium enrichment facility in Iran and is believed to have caused its production to drop by 15% in 2009 [1]. Obviously, security problems will cause a serious impact to the IoT.

As one of the key technologies involved in providing security, access control—determining* who* is allowed access,* when* access is permitted, and* where* access takes place—has been widely studied [2]. Access control models, which have been widely used, include role-based access control (RBAC) and usage control (UCON) [3, 4], Internet content control (ICCON) [5], Attribute-based access control [6], and user-driven access control [7].

Although these models succeed in the traditional Internet and operating systems, the IoT has raised several new and challenging issues surrounding the use of digital resources and its following critical characteristics make the above models not efficient any more. (1) Uncontrollable environments: sensors could be deployed in unattended environments, where physical nodes are more likely lost and false messages are more easily injected and transmitted. (2) Sensor-node resource constraints: computing and storage resources for sensor nodes are usually very limited, thereby severely constraining their ability to store and process the sensed data. Therefore high-weight access control models for the Internet should be revised for the sensing layer of the IoT. (3) Unreliable communications: the wireless communication adopted by the sensor nodes is often unreliable and unstable; therefore nodes may not receive the authorization in time. As a result, security in the IoT becomes more severe.

To minimize these threats, we proposed spatiotemporal access control based on reputation (STRAC), which considers time, location, and reputation as key elements in deciding whether access is granted or not. STRAC uses a lattice structure to decrease the storage complexity of policy bases. To reduce the risk caused by unreliable communications, we proposed nondeterministic authorizations (i.e., pessimistic, optimistic, and trade-off authorizations) and stochastic authorization. We demonstrate that pessimistic and trade-off authorizations are secure and that optimistic and stochastic authorizations can improve the QoS. In order to correctly update the reputation of nodes, we propose two novel policies (authority-based updates and election-based updates), based on the “group” characteristics of the sensing layer, and we prove that our proposed policies are secure. Our experiments show the efficiency of our model.

## 2. Related Work

Research about access control for the sensing layer can be divided into two general categories: access control algorithms (ACAs) and access control models (ACMs). ACAs mainly focus on new node addition. New node addition algorithms prevent malicious nodes from joining the sensor network. For example, [8] uses the self-certified elliptic curve Diffie-Hellman protocol to establish a pairwise key between new sensor nodes and the controller node, which launches a two-way authentication with the new nodes. However, in this scheme, all nodes share a network-wide key. Once one node is compromised, the secret key for all nodes must be updated, thereby causing huge losses. In order to solve this problem, [9, 10] proposes a new dynamic access control protocol, which uses hash functions to reduce computations and communications between two nodes.

In ACMs, much effort is spent on extending RBAC for pervasive computing. Reference [11] proposes a dynamic role-based access control (DRBAC) model, which provides context aware access control by dynamically adjusting role assignments and permission assignments based on context information. However, important features of the IoT (i.e., location and time) are not considered. In order to make RBAC more pervasive, many researchers extend RBAC by introducing time and location [12–15], where [12, 14] imposes spatiotemporal constraints on user-role assignments and permission assignments, and [15] introduces the concept of spatiotemporal zones and allows spatiotemporal constraints to be specified with prerequisite constraints. In addition, [16] adopts RBAC-based (role-based access control) authorization method using the thing's particular role(s) and application(s) in the associated IoT network. Reference [17] designs a capability-based access control delegation model for the federated IoT network. Reference [18] focuses on a minimal use of computation, energy, and storage resources at wireless sensors and proposes a novel access control solution for wireless network services in Internet of Things scenarios.

Although RBAC is often extended for pervasive computing, these extensions cannot be widely adopted for the sensing layer of the IoT because of the PSPACE-completeness [19] of RBAC.

Other spatiotemporal models that are not based on RBAC are also proposed. Reference [20] uses composition algebra to regulate access to patient data and balances the rigorous nature of traditional access control systems with the “delivery of care comes first” principle. Recently, reputation has been incorporated into models of access control for cyber-physical systems as in [21, 22]; however, these particular models do not deal with the loss of nodes.

Our work differs from the above solutions in several ways. First, we consider reputation, rather than roles, as a fundamental factor of access control for the sensing layer of the IoT, because the behavior of selfish nodes can be directly modeled by reputation but not easily modeled by roles. Such a change is nontrivial. If a node becomes selfish, then we are only required to assign a lower reputation to it. Therefore, reputation is more suitable than roles in controlling access to the sensing layer.

Second, the existing access control models do not efficiently handle the problem caused by unreliable communications. We propose nondeterministic authorizations and stochastic authorizations to solve this problem. Our method reduces the security risks of security-critical systems when failing to receive the key authorization instructions.

Finally, the existing models for the IoT do not consider the group characteristics of the sensing layer. In our work, node's reputation is cooperatively updated based on the group characteristics, thereby simplifying the reputation-update process.

## 3. Formalizing Time, Space, and Reputation

In order to construct a spatiotemporal access model based on reputation, we first formally define time, space, and reputation.

### 3.1. Reputation Description

Due to the limitations of storage and the computing resources, some nodes do not cooperate with others and demonstrate* selfishness*. In order to obtain more benefits, some nodes may attack others and demonstrate* misbehavior*. Because reputation (the opinion of one entity regarding another) can reflect both selfishness and misbehavior in interactions, it is adopted in modeling the behavior of nodes in our study.

Generally, from the aspect of reputation obtainment, reputation includes direct reputation (DR) and indirect reputation (IR), where DR and IR, respectively, refer reputation estimated by estimators based on their first-hand and second-hand experiences. From the aspect of goal, individual reputation should be distinguished with group reputation. Reference [23] surveys notions of reputation. In this paper, we only focus on individual and direct reputation, as follows.

We define reputation *REP* to have different ratings, and thus it can be denoted by a finite set; that is, *REP* = {*rep*_1_,…, *rep*_*n*_}, where *rep*_*i*_ is a reputation rating (1 ≤ *in*). Given any *rep*_*x*_ and *rep*_*y*_ in *REP*, they are mutually comparable, that is, *rep*_*x*_⪯*rep*_*y*_ or *rep*_*y*_⪯*rep*_*x*_. Thus, *REP* is a total order set. For a given node, its reputation is formed and ≤ updated through direct observations of its behavior and through feedback provided by other nodes. In this paper, we concentrate on general access models and do not discuss the methods of computing reputation in detail.

### 3.2. Time Description

In order to describe operations that can only be executed within a given time period, the notion of a calendar is adopted [24, 25]. A calendar consists of a countable set of contiguous intervals, for example, years, months, and days. Because two calendars can have different granularities, a subcalendar relationship can be established among them. That is, given two calendars *c*_1_ and, is a subcalendar of (written as *c*_1_⊑*c*_2_), if and only if there exists a natural number, such that *c*_2_ = *i* × *c*_1_. For example, days are a representative subcalendar of months. Obviously, ⊑ is a partial order relation. A calendar base *CB* represents a set of calendars and generally changes with different contexts. For example, if a school curriculum is comprised of years, semesters, and weeks, then its *CB* is {years, semesters, weeks}.

**Definition 1 (calendar time).**

Given *CB* = {*c*_1_,…, *c*_*n*_}, calendar time *ct* is defined as *ct* = ∑_*i*=1_^*n*^ *n*_*i*_ · *c*_*i*_, where, *n*_*i*_ ∈ *N* and for all 2 ≤ *i* ≤ *n*, one has *c*_*i*_⊑*c*_*i*−1_.

Let *CT* be a set of calendar times. Generally, any two calendar times are always comparable. That is, for any *ct*_*x*_ and *ct*_*y*_ in *CT*, one can have *ct*_*x*_ ≤ *ct*_*y*_ or *ct*_*y*_ ≤ *ct*_*x*_ (≤ is the total order relation). In the IoT, different types of time constraints exist, such as the earliest access time (*eat*), the latest access time (*lft*), the earliest finish time, and the latest finish time. In our study, *eat* and *lft* are adopted.

**Definition 2 (time constraints).**

Time constraint *TC*⊆*CT* × *CT* is a set of two-dimensional vectors of calendar times, where the first dimension and the second dimension represent *eat* and *lft*, respectively. Time constraints must satisfy the following condition: for any (*ct*_1_, *ct*_2_) ∈ *TC*, *ct*_1_ ≤ *ct*_2_.

**Example 3 .**

Given *TC* = {(*ct*_11_,  *ct*_12_), (*ct*_21_,  *ct*_22_)}, and an event satisfies *TC*, if access time (from start time to end time) of the event falls entirely within the time range from *ct*_11_ to *ct*_12_, or within the time range from *ct*_21_ to *ct*_22_.

A time constraint with overlapping ranges {(1,3), (2, 4)} can be reduced to {(1, 4)}, based on the following definition.

**Definition 4 (simplest time constraints).**

A time constraint *TC* is the simplest, if for any (*ct*_11_,  *ct*_12_) and (*ct*_21_,  *ct*_22_) ∈ *TC*, min⁡{*ct*_12_, *ct*_22_} < max⁡{*ct*_11_, *ct*_21_}.

**Figure 1:**
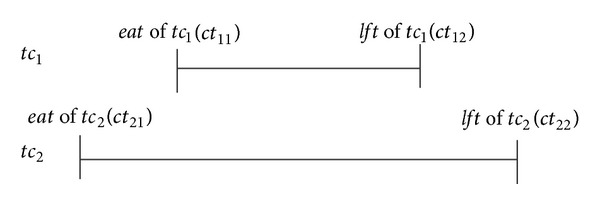
Time constraints.

Henceforth, one assumes that time constraints are always the simplest. Given two time constraints *tc*_1_ and *tc*_2_ shown in Figure 1, where (1) *ct*_11_—*eat* of *tc*_1_—is greater than or equal to that of *tc*_2_ and (2) *ct*_12_—*lft* of *tc*_1_—is less than or equal to that of *tc*_2_. If an event satisfies *tc*_1_, then it will satisfy *tc*_2_; this means that *tc*_1_ is stricter than *tc*_2_. Thus, one has Definition 5.

**Definition 5 (order relation ⪯ on *TC*).**

Given *tc*_1_ and *tc*_2_ in *TC*, *tc*_2_⪯*tc*_1_ (meaning that *tc*_1_ is stricter than *tc*_2_), if and only if *ct*_21_ ≤ *ct*_11_ and *ct*_12_ ≤ *ct*_22_, where *tc*_1_ = (*ct*_11_, *ct*_12_) and *tc*_2_ = (*ct*_21_, *ct*_22_).

**Proposition 6 .**

⪯ on *TC* is a partial order.

**Definition 7 (order relation ⪯ on 2^*TC*^).**

Given any *TC*_1_ and *TC*_2_, *TC*_1_⪯*TC*_2_  (meaning that *TC*_2_ is stricter than *TC*_1_), if and only if, for any *tc*_*x*_ ∈ *TC*_2_, there exists *tc*_*y*_ ∈ *TC*_1_ with *tc*_*y*_⪯*tc*_*x*_.

**Proposition 8 .**

⪯ on 2^*TC*^ is a partial order.

In the real environment, time constraints can be constructed by way of union or intersection of some constraints. One defines the intersection and the union as follows.

**Definition 9 (intersection).**

Intersection ⊙:2^*TC*^ × 2^*TC*^ → 2^*TC*^ is a function from 2^*TC*^ × 2^*TC*^ to 2^*TC*^ defined as

(1) $$\begin{array}{c} TC_{1}⊙TC_{2}=\left\{tc_{x}{⊙}_{TC}tc_{y}\;|\;tc_{x}\in TC_{1}\wedge tc_{y}\in TC_{2}\right\}, \end{array}$$

where

(2) $$\begin{array}{c} tc_{x}{⊙}_{TC}tc_{y}=\{\begin{array}{cc} \left(x,y\right) & \text{if}\;x\leq y\\ ⌀ & \text{otherwise}, \end{array}\\ tc_{x}=\left(ct_{x1},ct_{x2}\right),\;\;tc_{y}=\left(ct_{y1},ct_{y2}\right),\\ x=\max\left(ct_{x1},ct_{y1}\right),\;\;y=\min\left(ct_{x2},ct_{y2}\right). \end{array}$$

**Definition 10 (union).**

Union ⊕:2^*TC*^ × 2^*TC*^ → 2^*TC*^ is a function from 2^*TC*^ × 2^*TC*^ to 2^*TC*^ defined as *TC*_1_ ⊕ *TC*_2_ = {*tc*∣*tc* ∈ *TC*_1_∨*tc* ∈ *TC*_2_}.

Because the time constraints obtained by computing *TC*_1_ ⊕ *TC*_2_ are not always the simplest, one reduces them to the simplest form as follows. Given a time constraint *TC*, if there exists (*ct*_11_, *ct*_12_), (*ct*_21_, *ct*_22_) ∈ *TC* with max⁡{*ct*_11_, *ct*_21_} < min⁡{*ct*_12_, *ct*_22_}, then both (*ct*_11_, *ct*_12_) and (*ct*_21_, *ct*_22_) are deleted from *TC* and (min⁡{*ct*_11_, *ct*_2_ *lt*_1_1}, max⁡{*ct*_12_, *ct*_22_}) are inserted into *TC*. Obviously, the original *TC* is semantically equivalent to the modified *TC*. Henceforth, one assumes that the intersection and the union of time constraints are always the simplest.

**Proposition 11 .**

Given any *tc*_1_ and *tc*_2_ in *TC*, if *TC* is closed under ⊙ and ⊕, then *tc*_1_⊙*tc*_2_ and *tc*_1_ ⊕ *tc*_2_ are the supremum and the infimum of {*tc*_1_, *tc*_2_}, respectively.

The definitions above concentrate on physical time. However, in some cases, logical time (such as work time or class time) is more important.

**Definition 12 .**

*ti* *me* *As* *si* *gn* *ed*: *LT* → 2^*TC*^/{*⌀*} is a function mapping *LT* to the nonempty power set of *TC*, where *LT* = {*lt*_1_,…, *lt*_*n*_} represents a set of names of logical time.

**Definition 13 (order relation ⪯ on *LT*).**

Given any *lt*_1_ and *lt*_2_ in *LT*, *lt*_1_⪯*lt*_2_ if and only if *timeAssigned*(*lt*_1_)⪯*timeAssigned*(*lt*_2_).

**Proposition 14 .**

⪯ on *LT* is a partial order.

**Definition 15 (intersection on *LT*).**

(Here, we do not differentiate the ⊙ of Definition 9 from the ⊙ of Definition 15, because they are easily distinguished; Similarly, we also do not differentiate ⊕ of Definition 10 and ⊕ of Definition 16). ⊙:*LT* × *LT* → *LT* is an intersection function mapping *LT* × *LT* to *LT*, defined as *lt*_1_⊙*lt*_2_ = *lt*, where *timeAssigned*(*lt*) = {*x*⊙*y*∣*x* ∈ *timeAssigned*(*lt*_1_) and *y* ∈ *timeAssigned*(*lt*_2_)}.

**Definition 16 (union on *LT*).**

⊕:*LT* × *LT* → *LT* is a union function mapping *LT* × *LT* to *LT*, defined as *lt*_1_, where

(3) $$\begin{array}{c} \underset{\begin{array}{c} x\;\in \;timeAssigned(lt_{1})\\ y\;\in \;timeAssigned(lt_{2}) \end{array}}{⋃}x⊕y. \end{array}$$

**Proposition 17 .**

For any *lt*_1_ and *lt*_2_ in *LT*, if *LT* is closed under ⊙ and ⊕, then *lt*_1_⊙*lt*_2_ and *lt*_1_ ⊕ *lt*_2_ are the supremum and the infimum of {*lt*_1_, *lt*_2_}, respectively.

Proof that the supremum of {*lt*_1_, *lt*_2_} is *lt*_1_⊙*lt*_2_: from Definition 15, we have *lt*_1_⪯*lt*_1_⊙*lt*_2_ and *lt*_2_⪯⊙ *lt*_2_; therefore, *lt*_1_⊙*lt*_2_ is the upper boundary of {*lt*_1_, *lt*_2_}. Next, we prove that *lt*_1_⊙*lt*_2_ is the least element of the upper boundary of {*lt*_1_, *lt*_2_}. Let *lt*_1_⪯*lt* and *lt*_2_⪯*lt*; then, for any *x* ∈ *timeAssigned*(*lt*), there exists *y*_1_ ∈ *timeAssigned*(*lt*_1_) and *y*_2_ ∈ *timeAssigned*(*lt*_2_), such that *y*_1_⪯*x* and *y*_2_⪯*x*. According to Proposition 11, *y*_1_⊙*y*_2_⪯*x*. According to Definition 15, *lt*_1_⊙ *lt*_2_⪯*lt*; therefore, so *lt*_1_⊙ *lt*_2_ is the supremum of {*lt*_1_, *lt*_2_}.

### 3.3. Location Description

In the IoT, physical locations are often distinguished from logical locations. Physical locations are divided into two classes: hierarchical (topological, descriptive, or symbolic), such as a room, and Cartesian (coordinate, metric, or geometric), such as GPS position [12, 14, 26]. Logical locations represent the boundaries of the logical space that corresponds to the physical space.

Let *PLOC* = {*ploc*_1_,…,*ploc*_*n*_} be a set of physical locations, where *ploc*_*i*_  (1 ≤ *i* ≤ *n*) is a specific physical location, such as a 50 × 50 unit square area. Let *LLOC* = {*lloc*_1_,…,*lloc*_*n*_} represent the set of logical locations, where each in *LLOC* denotes the notion for one or more physical locations. Generally, relations between physical and logical locations are illustrated as a many-to-many map, denoted by *LP*⊆*PLOC* × *LLOC*.

**Definition 18 .**

A function *LlocToPloc*: *LLOC* → 2^*PLOC*^ maps *LLOC* to the power set of *PLOC*, returning all physical locations assigned to a given logical location. In other words, *c*(*lloc*) = {*ploc*∣(*ploc*, *lloc*) ∈ *LP*}.

**Definition 19 .**

A function *PlocToLloc*: *PLOC* → 2^*LLOC*^ maps *PLOC* to the power set of *LLOC*, returning all assigned logical locations of a given physical location. In other words, *c*(*ploc*) = {*lloc*∣(*ploc*, *lloc*) ∈ *LP*}.

Given two logical locations, a containment relation may exist. This is defined as follows.

**Definition 20 .**

A logical location *lloc*_*i*_ is contained in another logical location *lloc*_*j*_, written as *lloc*_*i*_⊆*lloc*_*j*_, if and only if *LlocToPloc*(*lloc*_*i*_)⊆*LlocToPloc*(*lloc*_*j*_).

Generally, physical locations of a given logical location are unchanged within a period; therefore, for simplicity, a logical location is used to denote its corresponding physical location. Similarly, one can define intersection ∩ and union ∪ based on logical locations, but one does not discuss them.

## 4. STRAC Framework

**Figure 2:**
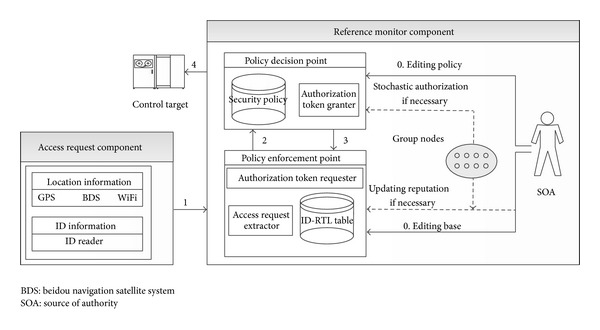
Framework of STRAC.

First, we provide an overview of the framework of our model. As shown in Figure 2, STRAC consists of two core components: the access request component (ARC) and the reference monitor component (RMC). The ARC of a node creates an access request, which has two forms: the first form includes five elements: the node's *ID*, the accessed object *o*, the expected operation *op* to be performed on *o*, the node's current location *ploc* (generally, a node's location information may come from GPSs or wifi), and the node's reputation *rep*; the second form includes three elements: the node's *ID*, the accessed object *o*, the expected operation *op* to be performed on *o*, and the node's current location *ploc*. In the first form, each node locally stores its reputation and physical location; in the second form, the reputation of each node is centrally stored in PEP (see the next paragraph for PEP) and its physical location is tracked by PEP.

RMC includes two modules: policy enforcement point (PEP) and policy decision point (PDP). The PEP module receives the user request, consults with the PDP module about the user authorization, and ensures that all access requests go through the PDP module. PEP is comprised of two submodules (access request extractor (ARE) and authorization token requester (ATR)) and two access tables which map the current time and physical locations to the logical time and the logical location, respectively (the two tables are called ID-RTL table in Figure 2). When the PEP module receives an access request from a user, ARE executes two steps: (1) it accepts the request and extracts the encapsulated location, *ID*, *o*, *rep*, *op*, and *ploc* if the first type of access requests is adopted; it extracts *ID*, *o*, and *ploc* if the second type is used, and (2) it queries the access database and returns the *ID*'s logical location *lloc* and logical time *lt* (in addition to the three elements, it returns the reputation of node *ID* if the second type of access requests is used). ATR encapsulatesthis information (*rep*, *lt*, *lloc*, *op*, and *o*) and sends it to the PDPmodule to request an authorization token (AT). Once this request is granted, an AT will be returned, and the user can access the target resources using this AT. PEP maintains a list of users' ATs, and this list is updated at a specified interval. AT will be revoked when the user deactivates the task or when the location and time associated with the use are out of the allowed scope.

The PDP module, comprised of one submodule (Authorization Token Granter (ATG)) and one policy base, makes the authorization decision based on a set of rules or policies. When PDP receives an AT request, it extracts the information (*rep*, *lt*, *lloc*, *op*, and *o*) from the request and consults the security policies. If the polices denote that a node with reputation *rep* at the time period *lt* in the location *lloc* has the right to perform the operation *op* on the target *o*, then ATG grants *AT* to access request.

The source of authority (SOA) is administrator or a group of administrators, who define the policies. SOA can also update the policy at runtime if necessary. In some cases, group nodes may also cooperatively update the node's reputation and make access decisions based on stochastic information (stochastic authorization in Figure 2).

Our model can be implemented with two alternative modes: ACI and AII. ACI focuses on authorization when complete information is available, while AII deals with authorization having only incomplete information. The precondition for ACI is that decision makers always can obtain authorization information in time. In other word, ACI requires a stable communication. Contrarily, this precondition is not necessary in AII. AII is very useful because unstable communications in the IoT are considered to be persuasive phenomenon. Generally, while in the ACI mode both PEP and PDP are mounted in the gateway and ARC is integrated into terminal nodes. Contrarily, to implement the AII mode, ARC and two lightweight modules, PEP and PDP, are mounted into terminal nodes.

## 5. STRAC Model

### 5.1. Basic Components of STRAC

The basic STRAC model is comprised of the following components:

 
*ID* = {*id*_1_,…,*id*_*n*_} is a set of node IDs;
 
*REP* = {*rep*_1_,…,*rep*_*n*_} is a set of reputations; for example, *REP* = {*g* *o* *l* *d* *rep* *u* *t* *a* *t* *i* *on*, *s* *il* *v* *e* *r* *rep* *u* *t* *a* *t* *i* *on*};
 
*LT* = {*lt*_1_,…,*lt*_*n*_} is a set of logical time constraints;
 
*LLOC* = {*lloc*_1_,…,*lloc*_*n*_} is a set of logical locations; for example, *LLOC* = {*a* *d* *m* *in* *i* *s* *t* *r* *a* *t* *o* *r* *r* *o* *o* *m*, *meetroom*};
 
*OP* = {*op*_1_,…,*op*_*n*_} is a set of operations; for example, OP = {*op* *e* *n*, *close*};
 
*O* = {*o*_1_,…,*o*_*n*_} is a set of target objects; for example, *O* = {*TV*, *Microwave*};
 
*PERM*⊆*OP* × *O* is a set of permissions, where (*op*,*o*) ∈ *PERM* means that *op* is performed on *o*. For example, if *PERM* = {(*op* *e* *n*, *TV*)} is assigned to device *id*, then the device owns the permission to open *TV*;
 
*AccZone*⊆*REP* × *LT* × *LLOC* is a set of *accesszones*, where each access zone is a triple (*rep*, *lt*, *lloc*);
 
*PA*⊆*PERM* × *AccZone* is a many-to-many map of connections between permissions and access zones. (*x*, *y*) ∈ *PA* means that any node with *y* has permission *x*. For example, ((*op*, *o*), (*rep*_1_, *lt*_1_, *lloc*_1_)) ∈ *PA* denotes that a node satisfying the constraint of *lt*_1_ with reputation *rep*_1_ at location *lloc*_1_ can execute operation *op* on object *o*;
 
*AccZone* *A* *s* *s* *i* *g* *n* *e* *d*: *PERM* → 2^*AccZone*^ assigns a permission level to access zones, where *AccZone* *A* *s* *s* *i* *g* *n* *e* *d*(*perm*) = {*AccZone*∣(*perm*, *AccZone*) ∈ *PA*}; that is, given a *perm*, function *AccZone* *A* *s* *s* *i* *g* *n* *e* *d* returns all access zones which the *perm* can access. For example, if *PA* = {((*op*_1_, *o*_2_), (*rep*_2_, *lt*_1_, *lloc*_2_)), ((*op*_2_, *o*_1_), (*rep*_2_, *lt*_2_, *lloc*_2_)), ((*op*_1_, *o*_2_), (*rep*_2_, *lt*_3_, *lloc*_1_))}, then *AccZone* *A* *s* *s* *i* *g* *n* *e* *d*((*op*_1_, *o*_2_)) = {(*rep*_2_, *lt*_1_, *lloc*_2_), (*rep*_2_, *lt*_3_, *lloc*_1_)};
 
*perm* *A* *s* *s* *i* *g* *n* *e* *d*: *AccZone* → 2^*PERM*^ assigns an access zone to permissions, where *perm* *A* *s* *s* *i* *g* *n* *e* *d*(*AccZone*) = {*perm*∣(*perm*, *AccZone*) ∈ *PA*}; that is, given an *acczone*, function *perm* *A* *s* *s* *i* *g* *n* *e* *d* returns all permissions by which the *acczone* can be accessed. For example, in the example of *AccZone* *A* *s* *s* *i* *g* *n* *e* *d*, *perm* *A* *s* *s* *i* *g* *n* *e* *d*((*rep*_2_, *lt*_1_, *lloc*_2_)) = {(*op*_1_, *o*_2_), (*op*_2_, *o*_1_)};
 
*AccessRequest*: *ID* × *OP* × *O* → {*true*, *false*} is a predicate; if it returns true, then node *id* requests permission to execute *op* on *o*. Recall that the storage of *rep* is either distributed or centralized. To model the two cases, we remove *rep* from access requests; for simplicity, we also remove the ID's location and reputation from access requests and encapsulate the three elements into the function *CurrentRTL* (we will discuss it next);
 
*AllowRequest*: *ID* × *OP* × *O*→{*true*, *false*} is a predicate; if it returns true, then node *id* is allowed to *op* on *o*;
 
*DenyRequest*: *ID* × *OP* × *O* → {*true*, *false*} is a predicate; if it returns true, then node *id* is not allowed to execute *op* on *o*;
 
*RevokeRequest*: *ID* × *OP* × *O* → {*true*, *false*} is a predicate; if it returns true, then the permission that node *id* executes *op* on *o* will be revoked;
 
*CurrentRTL*: *ID* → *REP* × *CT* × 2^*LLOC*^ is a function and returns *id*'s current reputation, its logical time, and logical locations (a node may be located in many different logical locations).

In order to return the logical locations of a node, its physical location must be first obtained, and then its logical locations can be computed using the function *PlocToLloc*. Because a physical location can be associated with many logical areas, *CurrentRTL* returns a set of logical locations.

### 5.2. Mechanism for Authorization and Revocation

Intuitively, if a node located in an appropriate area has an acceptable reputation and satisfies the given time constraints, its requests to execute some operations on an object should be allowed. In order to avoid using too many symbols, we overload the notation ∈, as follows.

Given *id*, *op*, and *o*, let *CurrentRTL*(*id*) = {*rep*_*id*_, *ct*_*id*_, {*lloc*_*id*1_,…, *lloc*_*idn*_}} and *AccZone* *A* *s* *s* *i* *g* *n* *e* *d*((*op*, *o*)) = {(*rep*_1_, *lt*_1_, *lloc*_1_),…, (*rep*_*n*_, *lt*_*n*_, *lloc*_*n*_)}; *CurrentRTL*(*id*) ∈ *AccZone* *A* *s* *s* *i* *g* *n* *e* *d*((*op*, *o*)) if and only if there exists (*rep*_*i*_, *lt*_*i*_, *lloc*_*i*_)*∈* *AccZone* *A* *s* *s* *i* *g* *n* *e* *d*((*op*, *o*)) with *rep*_*id*_ = *rep*_*i*_,  *ct*_*id*_ ∈ *lt*_*i*_ (*ct*_*id*_ ∈ *lt*_*i*_ if and only if (*ct*_1_, *ct*_2_) ∈ *timeAssigned*(*lt*_*i*_) holds, where *ct*_1_ ≤ *ct*_*id*_ ≤ *ct*_2_) and *lloc* *i* ∈ {*lloc*_*id*1_,…, *lloc*_*idn*_}. The authorization schemes are as follows:

1. *AccessRequest*(*id*, *op*, *o*)∧*CurrentRTL*(*id*) ∈ *AccZone* *A* *s* *s* *i* *g* *n* *e* *d*((*op*, *o*)) → *AllowRequest*(*id*, *op*, *o*)
2. *AccessRequest*(*id*, *op*, *o*)∧*CurrentRTL*(*id*) ∉ *AccZone* *A* *s* *s* *i* *g* *n* *e* *d*((*op*, *o*)) → *DenyRequest*(*id*, *op*, *o*)
3. *AllowRequest*(*id*, *op*, *o*)∧*CurrentRTL*(*id*) ∉ *AccZone* *A* *s* *s* *i* *g* *n* *e* *d*((*op*, *o*)) → *RevokeRequest*(*id*, *op*, *o*).

Formulas (1) and (2) show the following: (1) if the node *id* requests permission to execute *op* on *o*, and if *CurrentRTL*(*id*) (the current reputation, access time, and location of *id*) satisfies the conditions to execute *op* on *o*, then the request will be allowed; (2) if the node *id* requests permission to execute *op* on *o*, but *CurrentRTL*(*id*) does not satisfy the conditions to execute *op* on *o*, then the request will be denied. Formula (3) suggests that if node *id* has received the permission of executing *op* on *o* and *CurrentRTL*(*id*) no longer satisfies the conditions to execute *op* on *o* longer, the permission will be revoked.

## 6. Access Lattice

Because terminal nodes could move into many areas at different times, enumerating all areas and periods of time rapidly increases the size of PA (as shown above, PA connections between permissions and the power set of access zones). As a result, the size of the PA table could exceed the storage capacity. In addition, querying a big table consumes more energy and computing resources, thereby decreasing the efficiency of queries and even reducing a node's lifetime. Thus, decreasing the size of permission access table is critical. In order to achieve this goal, we adopted the access lattice in this study.

We make the following realistic assumptions regarding the sensing layer of the IoT. (1) A node with a high reputation can be granted all permissions of a lower-reputation node. (2) If a task can be executed in a wide area or a longer time period, then it can be also executed in a narrow area or a shorter time period. These assumptions mean that if one node owns two access zones *A* and *B*, where *A* is stricter than *B*, then *A* can be omitted from the set of access zones, because any permission allowed under *B* is allowed under *A*. We chose the lattice to decrease the size of the permission access table, because it models the strict relationship among elements. In order to formally describe the access lattice, we first define the order relation.

**Definition 21 (*order* *relation* ≤ *on* *AccZone*).**

Given any (*rep*_1_, *lt*_1_, *lloc*_1_) and (*rep*_2_, *lt*_2_, *lloc*_2_) ∈ *AccZone*, (*rep*_1_, *lt*_1_, *lloc*_1_)≤(*rep*_2_, *lt*_2_, *lloc*_2_), if and only if *rep*_1_⪯*rep*_2_, *lt*_1_⪯*lt*_2_, *lloc*_2_⊆*lloc*_1_.

**Theorem 22 .**

If (1) *LT* is closed under ⊙ and ⊕ and (2) *LLOC* is closed under ∩ and ∪, then (*AC* *CB* *A* *S* *E*, ≤) is a lattice.

Proof that there exists a supremum and an infimum for any (*rep*_1_, *lt*_1_, *lloc*_1_), (*rep*_2_, *lt*_2_, *lloc*_2_) ∈ *AC* *CB* *A* *S* *E*: if (*rep*_1_, *lt*_1_, *lloc*_1_) and (*rep*_2_, *lt*_2_, *lloc*_2_) are comparable, then there exists a supremum and an infimum for them. Even if (*rep*_1_, *lt*_1_, *lloc*_1_) and (*rep*_2_, *lt*_2_, *lloc*_2_) are incomparable, there exists a supremum and an infimum for them. Because ⪯ on *REP* is a total order, there exists a supremum for {*rep*_1_, *rep*_2_}; let the supremum be *rep*_*x*_, because *LT* is closed under both ⊙ and ⊕, and *LOC* is closed under ∩ and ∪; therefore, (*rep*_*x*_, *lt*_1_⊙*lt*_2_, *lloc*_1_∩*lloc*_2_) is in *AccZone* and is the upper boundary of (*rep*_1_, *lt*_1_, *lloc*_1_) and (*rep*_2_, *lt*_2_, *lloc*_2_). Let (*rep*, *lt*, *lloc*) be another upper boundary of (*rep*_1_, *lt*_1_, *lloc*_1_) and (*rep*_2_, *lt*_2_, *lloc*_2_), then we have *loc*⊆*lloc*_1_ and *loc*⊆*loc*_2_; therefore, *lloc*⊆*lloc*_1_∩*lloc*_2_. Because *rep*_*x*_ is the supremum of {*rep*_1_, *rep*_2_}, *rep*_*x*_⪯*rep*. According to Proposition 17, we have *lt*⪯*lt*_1_⊙*lt*_2_. Thus, (*rep*_*x*_, *lt*_1_⊙*lt*_2_, *lloc*_1_∩*lloc*_2_)⪯(*rep*, *lt*, *lloc*). Therefore, (*rep*_*x*_, *lt*_1_⊙*lt*_2_, *lloc*_1_∩*lloc*_2_) is a supremum of (*rep*_1_, *lt*_1_, *lloc*_1_) and (*rep*_2_, *lt*_2_, *lloc*_2_). Similarly, (*rep*_*y*_, *lt*_1_ ⊕ *lt*_2_, *lloc*_1_ ∪ *lloc*_2_) is an infimum of (*rep*_1_, *lt*_1_, *lloc*_1_) and (*rep*_2_, *lt*_2_, *lloc*_2_), where *rep*_*y*_ is an infimum of {*rep*_1_, *rep*_2_}.

We assume that *LT* is closed under ⊙  and ⊕, *LLOC* is closed under ∩ and ∪. We redefine the permission function under a lattice, as follows:

*pe* *rm* *As* *si* *gn* *ed*_lattice_: *AccZone* → 2^*PERM*^ maps the access zones to permissions, and *ermAssigned*_lattice_  (*AccZone*) = {*perm* ∈ *perm* *A* *s* *s* *i* *g* *n* *e* *d*(*AccZone*_*x*_)∣*AccZone*_*x*_⪯*AccZone*}.

Because *AccZone* *A* *s* *s* *i* *g* *n* *e* *d*_lattice_ and *AccZone* *A* *s* *s* *i* *g* *n* *e* *d* are similar and easily distinguished from one another; therefore, *AccZone* *A* *s* *s* *i* *g* *n* *e* *d* is adopted to denote the two functions in the sequel. Similarly, *perm* *A* *s* *s* *i* *g* *n* *e* *d* is used to denote *perm* *A* *s* *s* *i* *g* *n* *e* *d*_lattice_.

**Theorem 23 .**

Given any (*rep*_1_, *lt*_1_, *lloc*_1_) and (*rep*_2_, *lt*_2_, *lloc*_2_) ∈ *AccZone*, if (*rep*_1_, *lt*_1_, *lloc*_1_)⪯(*rep*_2_, *lt*_2_, *lloc*_2_), then *perm* *A* *s* *s* *i* *g* *n* *e* *d*((*rep*_1_, *lt*_1_, *lloc*_1_))⊆*perm* *A* *s* *s* *i* *g* *n* *e* *d*((*rep*_2_, *lt*_2_, *lloc*_2_)).

**Theorem 24 .**

Consider the following.

If *AllowRequest*(*id*, *op*, *o*)∧*AccessRequest*(*id*′, *op*, *o*)∧*CurrentRTL*(*id*)⪯*CurrentRTL*(*id*′), then *AllowRequest*(*id*′, *op*, *o*).

If *DenyRequest*(*id*, *op*, *o*)∧*AccessRequest*(*id*′, *op*, *o*)∧*CurrentRTL*(*id*′)⪯*CurrentRTL*(*id*), then *DenyRequest*(*id*′, *op*, *o*).

If *RevokeRequest*(*id*, *op*, *o*)∧*AllowRequest*(*id*′, *op*, *o*)∧*CurrentRTL*(*id*′)⪯*CurrentRTL*(*id*), then *RevokeRequest*(*id*′, *op*, *o*).

Theorem 24 shows (1) if a node with a low reputation can execute *op* on *o*, then another node with a higher reputation is also able to perform the same operation; (2) if a node with a high reputation is unable to execute operation *op* on *o*, then a node with a lower reputation is also unable to do so; and (3) if access permissions are revoked from a node with a high reputation, then the corresponding permissions are also revoked from a node with a lower reputation.

The following example illustrates that the lattice can efficiently decrease the size of policy bases.

**Example 25 .**

Let *PERM* = {(*Open*, *MicroWave*), (*SetParameter*, *MicroWave*), (*close*, *MicroWave*)}, *AC* *CB* *S* *E* = {(*rep*_1_, *lt*_1_, *lloc*_1_), (*rep*_2_, *lt*_2_, *lloc*_2_), (*rep*_3_, *lt*_3_, *lloc*_3_), (*rep*_4_, *lt*_4_, *lloc*_4_)}. The access base for each permission is as follows: *AccZone* *A* *s* *s* *i* *g* *n* *e* *d*((*close*, *MicroWave*)) = {(*rep*_1_, *lt*_1_, *lloc*_1_), (*rep*_2_, *lt*_2_, *lloc*_2_), (*rep*_3_, *lt*_3_, *lloc*_3_), (*rep*_4_, *lt*_4_, *lloc*_4_)}, *AccZone* *A* *s* *s* *i* *g* *n* *e* *d*((*SetParameter*, *MicroWave*)) = {(*rep*_3_, *lt*_3_, *lloc*_3_), (*rep*_4_, *lt*_4_, *lloc*_4_)} and *AccZone* *A* *s* *s* *i* *g* *n* *e* *d*((*Open*, *MicroWave*)) = {(*rep*_4_, *lt*_4_, *lloc*_4_)}. From above, the cardinality of *AccZone* *A* *s* *s* *i* *g* *n* *e* *d*((*Close*, *MicroWave*)), *AccZone* *A* *s* *s* *i* *g* *n* *e* *d*((*SetParameter*, *MicroWave*)), and *AccZone* *A* *s* *s* *i* *g* *n* *e* *d*((*Open*, *MicroWave*)) is 4, 2, and 1, respectively.

**Figure 3:**
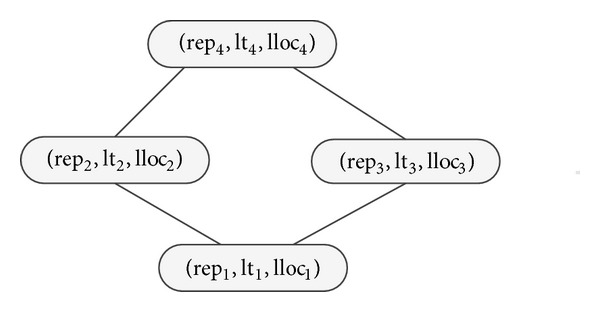
An access lattice.

Assuming that a lattice can be formed from these access zones as shown in Figure 3, then the access base for each permission could be changed as follows: *AccZone* *A* *s* *s* *i* *g* *n* *e* *d*((*close*, *MicroWave*)) = {(*rep*_1_, *lt*_1_, *lloc*_1_)}, *AccZone* *A* *s* *s* *i* *g* *n* *e* *d*((*SetParameter*, *MicroWave*)) = {(*rep*_3_, *lt*_3_, *lloc*_3_)}, and *AccZone* *A* *s* *s* *i* *g* *n* *e* *d*((*Open*, *MicroWave*)) = {(*rep*_4_, *lt*_4_, *lloc*_4_)}. This means that the cardinality of all three bases is 1. Given an access request *AccessRequest*(*id*, *Close*, *MicroWave*) from node *id* with reputation *rep*_3_ and assuming that node *id* is at the location *lloc*_3_ and the current time satisfies *lt*_3_, then *AllowRequest*(*id*, *Close*, *MicroWave*) will be true, because (*rep*_1_, *lt*_1_, *lloc*_1_)⪯(*rep*_3_, *lt*_3_, *lloc*_3_). From this example, we can deduce that the size of policy bases can be decreased using an access lattice.

Regarding the example given in the beginning of this section, if the above lattice is used, the storage complexity and the computing complexity are reduced to *n* and 1, respectively.

## 7. Authorization under Incomplete Information

In the above discussion, we mainly focused on authorization with complete information available (ACI), which is also called deterministic authorization. In other words, decision makers have the ability to obtain authorization information in time. However, this is not always the case. For example, when a node moves into a location where communication is unstable, it may not be able to obtain complete authorization information in time. In this case, decision makers have to choose whether to grant authorization or not, based on their own knowledge, such as historical experiences. This is related to authorization with incomplete information (AII). Lack of complete information presents the following challenges: (1) designing a secure authorization policy and (2) balancing security with QoS. To address these two challenges, we propose both nondeterministic authorizations and stochastic authorizations.

### 7.1. Nondeterministic Authorization

Nondeterministic authorization includes three alternative policies: pessimistic, optimistic, and compromise authorizations. Nodes run under the lowest permission levels in a pessimistic authorization, thus, providing only the most basic security.

**Policy 1 (pessimistic authorization).**

Given a access lattice formed by *AccZone* and an access request *AccessRequest*(*id*, *op*, *o*), if (*op*, *o*) ∈ *perm* *A* *s* *s* *i* *g* *n* *e* *d*(*glb*(*AccZone*)), then the request is allowed, where *glb*(*AccZone*) represents the greatest lower bound of set *AccZone*(i.e., the smallest elements of *AccZone*).

In order to execute Policy 1, all elements in *perm* *A* *s* *s* *i* *g* *n* *e* *d*(*glb*(*AccZone*)) must be locally stored. Although additional storage space is required by Policy 1, because both the greatest lower bounds of a lattice are unique, the storage complexity of the access rule table in Policy 1 is |*op*| × |*o*|; therefore, it is acceptable for the majority of weak-resources devices. Because glb(*AccZone*) is granted to the lowest permissions, Policy 1 is always secure (see Theorem 27 for the proof); however, this policy might have a lower QoS. For example, if the least permission is *ϕ* (empty), then any request will be automatically denied.

Contrary to pessimistic authorization, optimistic authorization concentrates on QoS while ignoring security. In this policy, nodes are granted the highest allowable permissions. From Theorem 22, *lub*(*AccZone*) is the largest element of *AccZone*, where *lub*(*AccZone*) represents the least upper boundary of *AccZone*. Thus, the greatest permissions are granted to *lub*(*AccZone*).

**Policy 2 (optimistic authorization).**

Given an access lattice formed by *AccZone* and an access request *AccessRequest*(*id*, *op*, *o*), if (*op*, *o*) ∈ *perm* *A* *s* *s* *i* *g* *n* *e* *d*(*lub*(*AccZone*)), then the request is allowed.

As with Policy 1, all elements of *perm* *A* *s* *s* *i* *g* *n* *e* *d*(*lub*(*AccZone*)) must be locally stored. Because any node in this policy is granted the greatest permissions, Policy 2 is not considered to be secure (see Example 28 for an example). However, this policy can efficiently improve QoS. For example, if the number of nodes with access to a film in digital rights management (DRM) is only optionally counted, any node can access this film anytime and anywhere, even when communications have been interrupted. If the film is stored locally, Policy 2 should be adopted, because security is not as much of an issue; if Policy 1 was used, the benefits surrounding optionally counting accesses cannot be achieved. Thus, Policy 2 has a higher QoS.

In many cases, security and QoS are needed to be balanced. To achieve this goal, we propose three compromise authorization policies: (1) trade-off authorization based on reputation (*TABR*), (2) trade-off authorization based on space (*TABS*), and (3) trade-off authorization based on time (*TABT*). We first discuss *TABR*.

Let *comp*_*rep*: *REP* → *AccZone* be a function from *REP* to *AccZone*, defined as *comp*_*rep*(*rep*) = *glb*{(*rep*_*x*_, *lt*, *lloc*) ∈ *AccZone*∣*rep*_*x*_ = *rep*}, representing the greatest lower boundary of access zones accessed by a node with reputation *rep*.

**Policy 3 (*TABR*).**

Given an access lattice formed by *AccZone* and an access request *AccessRequest*(*id*, *op*, *o*) in *AII*, if (*op*, *o*) ∈ *perm* *A* *s* *s* *i* *g* *n* *e* *d*(*comp*_*rep*(*rep*)), where *rep* is the reputation of node *id*, then the request is allowed.

Similarly, let *comp*_*loc*: *LLOC* → *AccZone* map from logical locations to *AccZone*, defined as *comp*_*loc*(*lloc*) = *glb*{(*rep*, *lt*, *lloc*_*x*_) *AccZone*∣*lloc*_*x*_ = *lloc*}, representing the greatest lower boundary of access zones accessed by a node in logical location *lloc*.

**Policy 4 (*TABS*).**

Given an access lattice formed by *AccZone* and access request *AccessRequest*(*id*, *op*, *o*) in *AII*, if (*op*, *o*) ∈ *perm* *A* *s* *s* *i* *g* *n* *e* *d*(*comp*_*loc*(*lloc*)), where *lloc* is the current logical location of node *id*, then the request is allowed.

Let *comp*_*lt*: *LT* → *AccZone* be a function from *LT* to *AccZone*, defined as *comp*_*lt*(*lt*) = *glb*{(*rep*_*x*_, *lt*, *lloc*) ∈ *AccZone*∣*lt*_*x*_ = *lt*}, representing the greatest lower boundary of access zones accessed by a node satisfying time constraint *lt*.

**Policy 5 (*TABT*).**

Given an access lattice formed by *AccZone* and an access request *AccessRequest*(*id*, *op*, *o*) in *AII*, if (*op*, *o*) ∈ *perm* *A* *s* *s* *i* *g* *n* *e* *d*(*comp*_*lt*(*lt*)), where *ct*() ∈ *lt*  (function *ct*: *ϕ* → *CB* returns the current time), then the request is allowed.

Because Policies 1–5 are related to incomplete information, their security must be formally analyzed.

**Definition 26 (security under *AII*).**

A policy is secure under *AII* if for any access request *AccessRequest*(*id*, *op*, *o*), *AllowRequest*(*id*, *op*, *o*) under *AII* is the same as *AllowRequest*(*id*, *op*, *o*) under *ACI*.

**Theorem 27 .**

Policy 1 and Policies 3–5 are secure.

Proof that Policy 1 is secure: given node id with reputation *rep* and an access request (*id*, *op*, *o*), because (*id*, *op*, *o*) is allowed under Policy 1, we have (*op*, *o*) ∈ *perm* *A* *s* *s* *i* *g* *n* *e* *d*(*lub*(*AccZone*)). Let (*rep*, *ct*(), *lloc*) be the reputation, the current time, and the logical location of node *id* under *ACI*, where (*rep*, *lt*, *lloc*) exists with (*rep*, *ct*(), *lloc*)∈(*rep*, *lt*, *lloc*) ((*rep*_1_, *ct*(), *lloc*_1_)∈(*rep*_2_, *lt*, *lloc*_2_), if *rep*_1_ = *rep*_2_ and *ct*() ∈ *lt* and *lloc*_1_ = *lloc*_2_). Because *glb*(*AccZone*) is the least element of *AccZone*, we have *glb*(*AccZone*)⪯(*rep*, *lt*, *lloc*). According to Policy 1 and Theorem 23, we have (*op*, *o*) ∈ *perm* *A* *s* *s* *i* *g* *n* *e* *d*(*glb*(*AccZone*))⊆*perm* *A* *s* *s* *i* *g* *n* *e* *d*((*rep*, *lt*, *lloc*)); therefore, (*rep*, *lt*, *lloc*) ∈ *AccZone* *s* *s* *i* *g* *n* *e* *d*((*op*, *o*)), *AllowRequest*(*id*, *op*, *o*) under *ACI* is true. That is, Policy 1 is secure.

Although Policy 1 and Policies 3–5 are secure, Policy 2 is insecure. An example is as follows.

**Example 28 .**

Let *AccZone* = (*rep*, *lt*, *loc*) with (*rep*, *lt*, *loc*)≺*lub*(*AccZone*). According to Theorem 23, there exists (*op*, *o*) with (*op*, *o*) ∈ *perm* *A* *s* *s* *i* *g* *n* *e* *d*(*lub*(*AccZone*))-*perm* *A* *s* *s* *i* *g* *n* *e* *d*((*rep*, *lt*, *loc*)). *AccessRequest* (*id*, *op*, *o*) will be allowed if Policy 2 is adopted; however, this request will be denied under *ACI*. This means that Policy 2 is insecure. Because security obeys the “leaky bucket” principle, Policy 2 is not suitable for security-critical systems.

### 7.2. Stochastic Authorization

In some cases, authorization may be considered to be stochastic. For example, when an automatically driven car arrives at crossroads, its central controller selects the road with the most gains (including both time and fuel-saving gains) by computing the distance to the destination and forecasting the probability of road congestion. In this case, road congestion is stochastic; as a result, the authorization is stochastic. Although stochastic authorization is actual requirement in the IoT, no efforts are spent on it in existing studies. In our study, we propose the (to the best of our knowledge) expectation-based authorization (EBA), where decision makers evaluate access requests by analyzing its potential gains in the successor states that would occur if the authorizations were granted. If the potential gains of the request are greater than or equal to a given threshold, it will be allowed. *EBA* includes the following elements besides the components mentioned in Section 5.1:

1. *S* = {*s*_1_,…, *s*_*n*_} is a set of states representing the potential successor states which the systems can arrive to after authorization.
2. *f*: *S* → *R* is a gain function, and *f*(*s*) represents the gain of decision makers in state *s*, *whereR* is a real number.
3. *p*: *S* → *R* is a probability distribution of state set *S*, and *p*(*s*) denotes the probability that systems would reach state *s*.
4. Threshold *r*.

The authorization rule is as follows: for any Access Request(*id*, *op*, *o*), if ∑_*s*=1_^*S*^ *p*(*s*) × *f*(*s*) ≥ *r*, then this request is granted. If several access requests exist and only one is allowed, then the request with the maximum gain is granted permission. Additionally, if multiple requests could have the maximum gain, then the decision maker randomly chooses among those requests.

As with Policy 2, expectation authorization improves the QoS. In the above example of automatic-driving systems, pessimistic authorization would deny any access request to each road, whereas optimistic authorization would allow all access requests for all roads. This is unacceptable for the automatic-driving systems. Thus, nondeterministic authorization (pessimistic/optimistic authorization) is unsuitable for automatic-driving systems. Conversely, because both the road environment and the historical experiences are taken into consideration to ensure that only the request with the maximum gain is allowed, stochastic authorization is suitable for automatic-driving systems.

## 8. Mechanism for Updating Reputation

Because nodes of the IoT are easily tampered with, the reputation of the tampered nodes must be updated in time. In this section, we borrow the idea of downgrading in programming languages [27] and propose a new mechanism to update the reputation for the sensing layer of the IoT. In this mechanism, every node is bound to an update policy and any change to a node's reputation must be consistent with the bound policy; that is, the reputation can be updated, only if a given policy for that node is satisfied. The syntax of the general update mechanism is defined in Table 1.

The above definitions are based on Backus Normal Form; for example, *rep*⊳_*c*_1__ *rep*2⊳_*c*_2__ *ϕ* is grammatically correct. In Table 1, if the empty policy (*ϕ*) is adopted for a node, then its reputation is preserved. Given ⊳_*c*_1__ *updatep* for a node means that if condition *c*_1_ is true, then the reputation of the node will be updated to *rep*_1_, and the successor of the policy is updated to *updatep*. The formal semantics of this mechanism are as follows:

(4) rulegeneral:cid:rep⊳cupdatep⟹id:updatep[rep/currentREP(id)]ruleϕ: id:⌀⟹⌀,

where *id* : *rep*⊳_*c*_ *updatep* represents the node *id* as bound to *rep*⊳_*c*_ *prenew* andfunction *c* *u* *r* *r* *e* *n* *t* *REP*: *ID* → *REP* returns the current reputation of a given node. [*rep*/*c* *u* *r* *r* *e* *n* *t* *REP*(*id*)] denotes that *c* *u* *r* *r* *e* *n* *t* *REP*(*id*) is substituted by *rep*. *rule*_*general*_ indicates that if the predicate *c* is true, then the current reputation of node *id* will be updated to *rep* and its new policy is *updatep*. *rule*_*ϕ*_ stops the update. Specifically, when the update policy for a node is *rep*⊳_*T*_ *updatep*, then its reputation will be mandatorily updated to *rep*. In contrary, if the policy is *rep*⊳_*F*_ *updatep*, then the update will be forbidden.

In Table 1, basic predicates are coarse-grained and difficult to use. In order to solve this problem, we propose two submechanisms: authority-based update mechanisms (*AUM*) and election-based update mechanisms (*EUM*).

### 8.1. Authority-Based Update Mechanisms (AUM)

In *AUM*, only the authority node can update the reputation of other nodes. A node is called an authority node if its reputation is greater than or equal to a given threshold *rep*_*auth*_, where *rep*_*auth*_ represents the authority reputation. To formally define semantics, we first provide the following definitions.

*cu* *rr* *en* *tT* *L*: *ID* → *CB*  2^*LLOC*^ returns the current time and the set of current logical locations for a given node.

*Up* *da* *te* *TL*⊆*LT* × *LLOC* is a subset of the product of *LT* and *LLOC*. A node is granted the update permission, only if its current time and one of its current logical locations belong to the set *UpdateTL*. In other words, node *id* can update the reputation of other nodes, only if *currentTL*(*id*) ∈ *UpdateTL*  (let *currentTL*(*id*) = (*cb*,{*lloc*_1_,…, *lloc*_*n*_}) and *UpdateTL* = {(*lt*_*i*1_, *lloc*_*i*1_),…,(*lt*_*im*_, *lloc*_*im*_)}, we define *currentTL*(*id*) ∈ *UpdateTL*, if and only if there exists (*lt*_*il*_, *lloc*_*il*_) ∈ *UpdateTL* such that *cb* ∈ *lt*_*il*_ and *lloc*_*k*_ = *lloc*_*il*_, *where*1 ≤ *k* ≤ *n*).

The formal semantics of AUM are as follows:

(5) ruleauthority: currentREP(idr)=repr currentTL(idr)∈UpdateTL rep,currentREP(id)≺repthershholdreprid:rep⊳authupdate(repr)updatep⟹ id:updatep[rep/currentREP(id)] ruleϕ: id:⌀⟹⌀.

The predicate *auth* *u* *p* *d* *a* *t* *e*(*rep*_*r*_) is true, if and only if there exists *id* ∈ *ID* with *c* *u* *r* *r* *e* *n* *t* *REP*(*id*_*r*_) = *rep*_*r*_,  *currentTL*(*id*_*r*_) ∈ *UpdateTL*, *rep*≺*rep*_*thershhold*_, *c* *u* *r* *r* *e* *n* *t* *REP*(*id*)≺  *rep*_*thershhold*_, and *rep*_*thershhold*_⪯*rep*_*r*_. The rule *rule*_*authority*_ states that the reputation of node *id* can be updated to *rep* if the following conditions are satisfied: (1) *rep*_*thershhold*_⪯  *c* *u* *r* *r* *e* *n* *t* *REP*(*id*_*r*_); that is, node *id*_*r*_ is an authority node. (2)  *currentTL*(*id*_*r*_) ∈ *UpdateTL*; that is, node *id*_*r*_ satisfies the temporal and spatial constraint. (3) The current reputation of node *id* and its reputation after update are both greater than or equal to *rep*_*threshold*_.

AUM can prevent a node's reputation from being illegally updated even if authority nodes are lost. For example, the authority node *x* in the location *lloc* can update the reputation of others. If *x* is lost, it may not be in the location *lloc*. Let *x* be in the location *loc*_*theft*_ with *loc*_*theft*_ ≠ *lloc*, therefore, making *currentTL*(*id*) ∉ *UpdateTL*. This means that *x* no longer has the ability to update the reputation of other nodes.

### 8.2. Election-Based Update Mechanisms (EUM)

Although *AUM* can be used to decrease the risk caused by the loss of nodes, authority nodes are security-critical because of the huge risks involved if they are compromised. In order to solve this problem, we propose *EUM*. In *EUM*, a group of nodes with a lower reputation are able to update the reputation of others through elections. When the majority of voters (nodes) agree on an update, the reputation of a specific node can be updated. In order to precisely define this mechanism, we first define the update function *f* : *ID* × *ID* × *REP* → {0,1}, which maps *ID* × *ID* × *REP* to {0,1}: If a voter *id* requests to update the reputation of candidate *id*_*c*_ to *rep*_*c*_, then *f*(*id*_*c*_, *id*, *rep*_*c*_) = 1. The formal semantics of *EUM* is as follows:

(6) ruleelection:Y≥ridc:repc⊳Y≥rupdatep⟹id:updatep[repc/currentREP(idc)],

where *Y* = ∑_*id*_^*ID*_*TL*_^ *f*(*id*_*c*_, *id*, *rep*_*c*_), *ID*_*TL*_ = {*x* ∈ *ID*∣*currentTL*(*x*) ∈ *UpdateTL* *currentTL*(*x*) ∈ *UpdateTL*}, and *r* is a natural number. *Rule*_*election*_ shows that if at least *r* nodes satisfying the given time constraints in the given areas request to update the reputation of node *id* to *rep*, then these requests will be approved and id's reputation will be updated. Because *rule*_*election*_ can update the reputation of any other node, *r* must be carefully defined, especially in networks where malicious nodes are dominant. Generally, *r* could be equal to ⌈*N*/2⌉ or ⌈2*N*/3⌉, where *N* is the number of voters.

**Example 29 .**

Let the update policy of node *id* be *rep*_0_  ⊳_*authupdate*(*rep*_*r*_)_ *rep*_1_⊳_*T*_ *ϕ*; we have the following results.

1. If the current reputation of *id* is greater than *rep*_*threshold*_, then the reputation of *x* will be preserved.
2. If authority node *id*_*r*_, which is not located in the given areas or does not satisfy the given time constraints (i.e., *currentTL*(*id*_*r*_) ∉ *UpdateTL*), requests to update the reputation of node *id* will be denied and the reputation of *id* will be preserved.
3. If the current reputation of *id* is less than *rep*_*threshod*_ and authority node *id*_*x*_ requests to update the reputation of node *id*, where *currentTL*(*id*_*x*_) ∈ *UpdateTL* and *rep*_0_⪯*rep*_*x*_, then this request will be approved and the reputation of *id* will be updated to *rep*_0_.

**Example 30 .**

Let the update policy of node *y* be *rep*_0_⊳_*Y*≥10_ *rep*_1_⊳_*T*_ *ϕ* and let its current reputation be *rep*_0_. In this case, if at least 10 nodes at a given period and location send requests to update the reputation of *x* to *rep*_0_, then the reputation will be updated.

### 8.3. Order Relation

It is possible that a single node owns two update policies, with one policy being stricter than the other. In this case, an order relation can be constructed to decrease the consumption of computing resources. Let ≤_*p*_ denote that is stricter than; that is, if can be adopted, then can be used. ≤_*p*_ is recursively defined as follows (note that only *rule*_*authority*_ is considered here):

(7) ≤pa1:ϕ≤pupdatep≤pa2:rep1⪯rep2updatep1≤pupdatep2rep1⊳authupdate(repr)updatep1≤prep2⊳authupdate(repr)updatep2.

≤_*pa*_1 shows that the empty policy is the loosest because it can be used anywhere and anytime; ≤_*pa*_2 denotes that if (1)  *rep*_2_ is greater than or equal to *rep*_1_ and (2)  *updatep*_2_ is stricter than *updatep*_1_, then *rep*_2_⊳_*authupdate*(*rep*_*r*_)_ *updatep*_2_ is stricter than *rep*_1_⊳_*authupdate*(*rep*_*r*_)_ *updatep*_1_.

To study the properties of ≤_*p*_, we define the necessary function *first*, which maps policy to the reputation, as follows:

(8) $$\begin{array}{c} first\left(prenew\right)=\{\begin{array}{cc} rep & \text{if}\;\;prenew\equiv rep\;{⊳}_{c}\;{prenew}^{\prime }\\ ⌀ & \text{otherwise}. \end{array} \end{array}$$

**Proposition 31 .**

If *updatep*_1_≤_*p*_ *updatep*_2_, then first(*updatep*_1_)⪯*first*(*updatep*_2_).

**Proposition 32 .**

If *updatep*_1_≤_*p*_ *updatep*_2_ and *rep*_1_⪯*rep*_2_, then *updatep*_1_[*rep*_1_/*first*(*updatep*_1_)]≤_*p*_ *updatep*_2_  [*rep*_2_/*first*(*updatep*_2_)].

**Theorem 33 .**

(*P*, ≤_*p*_) is a total order, where *P* is a set of policies.

**Theorem 34 .**

For any nonempty policies *updatep*_1_ and *updatep*_2_, such that *updatep*_1_≤_*p*_ *updatep*_2_, if there exists a nonempty policy *updatep*_*x*_ with *id*: *updatep*_2_⇒*id*: *updatep*_*x*_, then there also exists a nonempty policy *updatep*_*y*_ with *id*: *updatep*_1_⇒*id*: *updatep*_*y*_ and *updatep*_*y*_≤_*p*_ *updatep*_*x*_.

**Proof**

Let *updatep*_2_ be *p*_20_⊳_*authupdate*(*rep*_*r*_)_ *updatep*_*x*_. From *id* : *updatep*_2_⇒*id* : *updatep*_*x*_ and rule_authority_, we have *id*_*r*_ ∈ *ID* with *c* *u* *r* *r* *e* *n* *t* *REP*(*id*_*r*_) = *rep*_*r*_, *currentTL*(*id*_*r*_) ∈ *UpdateTL*, *rep*≺*rep*_*thershhold*_, *c* *u* *r* *r* *e* *n* *t* *REP*(*id*)≺*rep*_*thershhold*_, and *rep*_*thershhold*_⪯*c* *u* *r* *r* *e* *n* *t* *REP*(*id*_*r*_). Let *updatep*_1_ be *p*_10_⊳_*authupdate*(*rep*_*r*_)_ *updatep*_*yi*_. Because *updatep*_1_≤_*p*_ *updatep*_2_, we have *rep*_10_⪯*rep*_20_ and *updatep*_*yi*_≤_*p*_ *updatep*_*x*_. Because *rule*_*authority*_, we have *id* : *updatep*_1_⇒*id* : *updatep*_*yi*_[*rep*_10_/*c* *u* *r* *r* *e* *n* *t* *REP*(*id*)]. From Proposition 32, we have *updatep*_*y*_≤_*p*_ *updatep*_*x*_, where *updatep*_*y*_ = *updatep*_*yi*_[*rep*_10_/*c* *u* *r* *r* *e* *n* *t* *REP*(*id*)].

## 9. Verification of Security Policy 

The STRAC model has many features that could interact with each other, causing conflict and inconsistency between security policies. As a result, security policies must be verified before they are applied. Tediousness and proneness of manual analyses make automatic verification necessary. UPPAAL [28] is an integrated model checker for modeling, validation, and verification of real-time systems modeled as networks of timed automata. In this study, UPPAAL is used to verify whether the policy conforms to security requirements or not. When requirements are violated, the tool pictorially shows how the property has been violated and generates a counterexample to help security designers fix the policy.

To illustrate how to formally specify and verify a STRAC policy, we consider smart home applications. We assume the existence of four smart devices in this example: (1) a TV set (*TV*), (2) an air conditioning (*AC*) unit, (3) a microwave (*MW*), and (4) an electric rice cooker (*RC*). Each of these devices can be remotely controlled using mobile terminals. We also stipulate that parents and their children can use these devices only in the office, school, and home. In order to provide the necessary security for these devices, we make the following security policies: (1) the TV set and the *AC* can be closed or opened either remotely or locally because of the low heat produced by such devices; (2) the *RC* can only be opened locally but can be closed remotely because of the high degree of heat that it produces; (3) the *MW* can be opened by parents either remotely or locally but cannot be opened by children remotely, because of the heat produced by the *MW* and the need to configure a time parameter, which can be performed only by parents; (4) because of the limitations regarding power load, the maximum number of devices that is able to run simultaneously is set to three; (5) every device only runs during the specified time.

### 9.1. Model

We integrate the components of STRAC as follows: *ID* = {0,1, 2} is a set of mobile terminal IDs based on the assumptions that (1) Terminal 0 is owned by children and is used in school and at home, (2) Terminals 1 and 2 are owned by parents and are used in multiple locations (school, office, and home), and (3)  *REP* = {*highRep*, *lowRep*}, where *highRep* and *lowRep* denote high reputation and low reputation, respectively. We assume that the reputation of Terminal 0 is low and the others are high. *LT* = {*TV* *t* *im* *e*, *AC* *t* *im* *e*, *MW* *t* *im* *e*, *RC* *t* *im* *e*} is a set of logic constraint times, for operating *TV*, *AC*, *MW*, and *RC*. *LLOC* = {*home*, *office*, *school*}, *OP* = {*op* *e* *n*, *close*, *c* *on* *f* *i* *g*}, *O* = {*TV*, *AC*, *MW*, *RC*}, and *PERM* = {*op* *e* *n*, *close*} × *O* ∪ {(*c* *on* *f* *i* *g*, *MW*)}; *AccZone* is the product of *REP*, *LT*, and *LOC*. *PA* is a core component determined by control policies, as shown in Table 2. In Table 2, the product of the left and right columns of any row is an element of *PA*. For example, {(*op* *e* *n*, *TV*)} × *REP* × {*TV* *t* *im* *e*} × *LLOC*⊆*PA*. According to Table 2, *AccZone* *A* *s* *s* *i* *g* *n* *e* *d* and *perm* *A* *s* *s* *i* *g* *n* *e* *d* can be obtained (not discussed in this paper). For any access request, a decision can be made. For example, a child requesting to open *MW* in *school* will be denied.

### 9.2. Policy Verification

In the home applications discussed above, three kinds of entities exist: mobile terminals, RM (as shown in Section, RM denotes reference monitor), and the related devices (*TV*, *AC*, *MW*, and *RC*). In UPPAAL, these entities are denoted as *M* *o* *b* *il* *e* *T* *e* *rm*, *RM*, and *Device*, respectively. In this scenario, mobile terminals send access requests to the RM. Upon receiving these requests, RM retrieves the stored access rule table, decides whether to approve these requests, and sends the result to the controlled devices.

**Figure 4:**
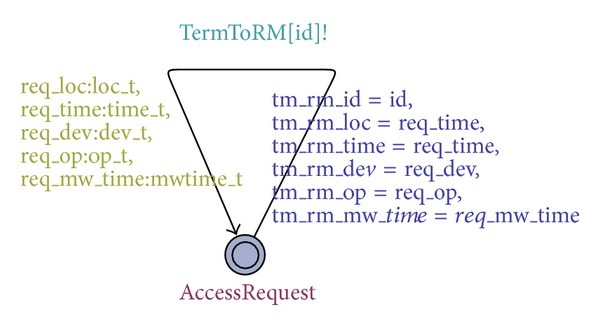
Timed automata of mobile terminals.

An access request consists of six parameters (as shown in Section 3.1, only three parameters are needed; however, in UPPAAL, we cannot get the current time; therefore, the *t* *im* *e* parameter is necessary. For simplicity, both *loc* *a* *t* *i* *on* and *t* *im* *e* are provided here): *tm*_*rm*_*id* (ID of the mobile terminals), *tm*_*rm*_*loc* (location of the mobile terminals), tm_rm_time (current time), *tm*_*rm*_*dev* (target device), *tm*_*rm*_*op* (operation to perform on the target device), and *tm*_*rm*_*MW*_*t* *im* *e* (timer for *MW*).  Figure 4 gives the timed automata of mobile terminals, where *id* is an input parameter, *T* *e* *rm* *T* *o* *RM*[*id*] is a channel, and *loc*_*t* is a set of all locations (*home*, *school*, and *office*). The remaining variables are similar to *loc*_*t*.

**Figure 5:**
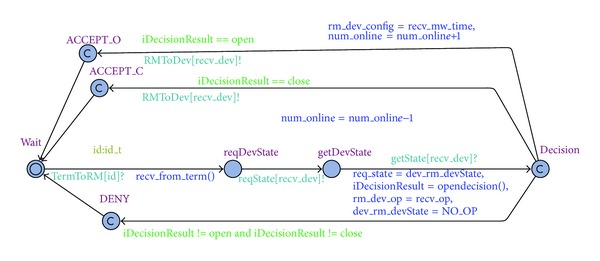
Timed automata of RM.

As shown in Figure 5, *RM* receives access requests from the channel *T* *e* *rm* *T* *o* *RM*[*id*], checks the state (busy or free) of the controlled device, and makes a decision according to the reputation and location of the mobile terminals and the current time. This decision is then sent to the corresponding device.

**Figure 6:**
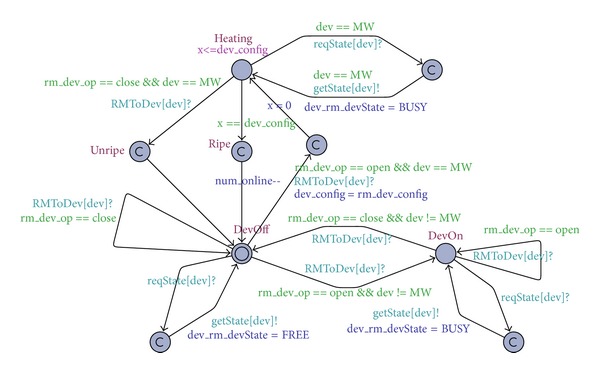
Timed automata of controlled devices.

Figure 6 illustrates the timed automata of controlled devices. The initial state of each device is DevOff. When the instruction *op* *e* *n* arrives, the corresponding device starts to run. If the instruction *close* arrives, the corresponding device stops running. In *MW*, *dev*_*c* *on* *f* *i* *g* is used to store the time parameter of the *MW* timer.

The composition (MobileTerm ||RM|| Device) of the above three timed automata forms a timed automation network, and we can use this network to verify policies. In our studies, three properties are verified: (1) “deadlock must be avoided in smart home applications.” This can be specified as “A[] not deadlock” by using computation tree logic (CTL); (2) the number of simultaneously running devices is always less than or equal to three. This can be specified as “A[] not (Device(TV).DevOn and Device(AC).DevOn and Device(RC).Heating and Device(*MW*).DevOn)”; (3) when the parents open a microwave in office, the microwave would always switch to the *Ripe* state, which is “(tm_rm_id==1&&tm_rm_loc==office&& tm_rm_time==8&&tm_rm_dev==*MW*&& tm_rm_op== open) –>Device(*MW*).Ripe,” where the reputation of Terminal 1 (belonging to the parents) is high and the current time (m_rm_time==8) is within the work time of *MW*.

We use UPPAAL to verify the three properties, and the result shows that the first two properties are true. This means that the system is deadlock-free and that the number of simultaneously running devices is always less than or equal to three. Property (3) is proven to be false, meaning that, even when parents start the microwave while in the office, the microwave does not necessarily switch to the *Ripe* state. A counterexample is as follows: if parents start the microwave and the rice cooker at the same time in the office, the child will not be able to start the air conditioning and watch TV due to the power load limitation. In this case, children could stop the microwave, thus preventing it from switching to the *Ripe* state.

## 10. Experiments

**Figure 7:**
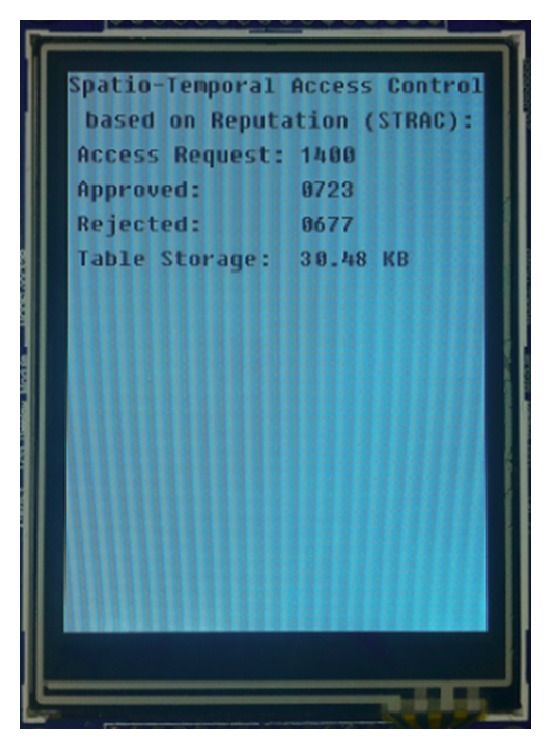
STRAC prototype.

We develop a prototype (shown in Figure 7) that implements STRAC. In our prototype, we use a network topology consisting of 10 terminal nodes (TeNs) uniformly deployed in a × area with TeNs surrounding a sink node (SiN), where Texas Instruments CC2530 chips with 8-KB RAM and 256-KB programmable flash are adopted for the TeNs and an ST Microelectronics STM32 chip with 64-KB RAM and 512-KB programmable flash was used for the SiN (CC2530 and STM32 chips are widely used as wireless transceivers and data processors in industry). The ZigBee protocol is used for communications between the TeNs and the SiN. The ARC component is mounted on the TeNs while RMC components are mounted on the SiN. ARC and RMC are written as traditional *C* programs.

Generally, heavyweight database servers, such as MySQL, are not appropriate for terminal nodes because of their limitations in storage and computing resources. To solve this problem, we implement a table query algorithm in our prototype and we also design two database tables: an ID-RTL table that maps node ID to an RTL triple (the reputation of the node, the current logic time, and the current time location) and an access rule table that stores PA (as shown in Section 5, PA connects permissions with access zones). The ID-RTL table is integrated into the PEP components and is only accessible by TeNs and PDP, while the access rule table is mounted into PDP components and is only accessible by PEP. Our experiment setup is as follows: (1) reputation is set to 5 ratings, (2) TeNs could move into 100 different areas at 10 logical time periods, and (3) TeNs may perform 3 operations on 50 objects. After *PA* is explicitly defined, a series of experiments are conducted. The results reported in this section are averaged over 10 runs.

**Experiment 1 (correctness of implementation).**

To evaluate correctness of implementation, we create two groups (*A* and *B*) of access requests in two local files: any request of Group *A* is in the access rule table; that is, any request from should be approved; all elements in Group are out of the access rule table; that is, any request from should be rejected. Groups *A* and *B* allow us to test whether our prototype works as anticipated. The experiment result shows that all requests from group *A* are approved and all requests from group *B* are rejected. This fact demonstrates the correctness of our implementation.

**Experiment 2 (storage resources).**

In this experiment, two cases are considered in *PA* one is “given one permission, the number of its access zones is randomly generated” (called random scheme); the other is “given one permission, the number of its access zones is constant” (called constant scheme). The experiment result is as follows.

*Random Scheme*. If PA is compiled with the nonlattice strategy, 7500 access rules occupy 36.5 KB of programmable flash; however, if the lattice strategy is adopted, only 3750 access rules are needed; as a result, 36.62 KB of programmable flash is consumed, which is about 100.3% of memory used by the nonlattice strategy.

*Constant Scheme*. If PA is compiled with the nonlattice strategy, 15000 access rules occupy 73.24 KB of programmable flash; however, if the lattice strategy is adopted, only 3900 access rules are needed; as a result, 30.48 KB of programmable flash is consumed, which is about 41.6% of memory used by the nonlattice strategy.

*Note*. In this experiment, PA is stored via a two-dimensional array. This means that for any two permissions, their consumed storages are the same and equal to *sizeof* (*array*)/*length* (*array*). As a result, in the random scheme,the storage occupied by the lattice strategy is slightly greater than that of the lattice strategy. If PA is stored via files or pointers, the occupied storage will approach the constant scheme.

**Experiment 3 (average response time, *ART*).**

To evaluate the response time of our scheme, we add a Group *C* of access requests: almost half of the access requests in Group *C* are in the access rule table. Each TeN continuously sends 1400 access requests to the SeN. The experiments show that the *ART*, which is the average delay from receiving a request to returning the response, is 21.4 ms if the nonlattice policy is adopted. The *ART* is reduced to 18.6 ms if the lattice policy is used, which is about 86.9% of the time used by the nonlattice strategy.

Experiments 2 and 3 show that the lattice policy is better overall than nonlattice policy and that the storage resource consumed and the response time of our model are acceptable for the sensing layer of the IoT.

## 11. Conclusions

The IoT presents new types of architectures, vulnerabilities, and requirements. Consequently, existing access control models have to be revised to accommodate these changes. Although spatiotemporal access models have been proposed in previous studies, most of them ignore some important characteristics of the sensing layer of the IoT. In this paper, we abstract the basic characteristics of the IoT's sensing layer and propose a model (called STRAC) that combines space and time with reputation for access control of the IoT's sensing layer. Our model solves the problem of deciding *when* and *where* to authorize access requests and *who* is able to access information by using spatial/temporal information, and it uses nondeterministic/stochastic authorizations to deal with unstable communications. These methods either provide better security or improve the QoS. In order to more precisely manage the reputation of nodes, we present a novel mechanism to update reputation and demonstrate its security. STRAC overcomes the inadequacies of existing access controls while acting as an access control foundation for the sensing layer of the IoT. In future work, we will design a scheme to find the optimal trade-off between security and QoS.

## Figures and Tables

**Table 1:** Syntax of update policies.

*updatep*::=	Policies
*re* *p*⊳_*c*_ *updatep*	Update policy
*ϕ*	Empty policy
*c*::=	Condition
Basic predicate	Basic predicate
T	True
F	False
*c*∨*c*	Disjunction
¬*c*	Negation

**Table 2:** *PA* of smart home applications.

PERM	Access zones
(*open, TV*)	*RE* *P* × {*TV* *t* *im* *e*} × *LLOC*
(*open, AC*)	*RE* *P* × {*AC* *t* *im* *e*} × *LLOC*
(*open, MW*)	{*highRep*}×{*MW* *t* *im* *e*} × *LLOC*
(*config, MW*)	{*highRep*}×{*MW* *t* *im* *e*} × *LLOC*
(*open, RC*)	*RE* *P* × {*RC* *t* *im* *e*}×{*home*}
{*close*} × *O*	*RE* *P* × *LT* × *LLOC*
